# Environmental radiation assessment in the specified living areas for returnees of Okuma Town, Fukushima prefecture: Field measurements prior to decontamination

**DOI:** 10.1371/journal.pone.0354457

**Published:** 2026-07-24

**Authors:** Kyoka Majima, Yuya Kashiwazaki, Hitomi Matsunaga, Makiko Orita, Noboru Takamura

**Affiliations:** 1 Department of Disaster Resilience and Science, Nagasaki University Graduate School of Biomedical Sciences, Nagasaki, Japan; 2 The Great East Japan Earthquake and Nuclear Disaster Memorial Museum, Fukushima, Japan; Japan Atomic Energy Agency / Ibaraki University, JAPAN

## Abstract

To assess environmental radiation exposure in the Specified Living Areas for Returnees of Okuma Town, Fukushima Prefecture, external radiation doses were evaluated using ambient dose equivalent rate measurements performed at residential properties. In addition to surveys covering the entirety of the Specified Living Areas for Returnees, area-specific assessments were conducted to account for differences in the progress of decontamination and demolition among districts. The median ambient dose rate measured by car-borne surveys across all Specified Living Areas for Returnees of Okuma Town decreased significantly from 0.49 µSv/h in November 2024 to 0.34 µSv/h in September 2025 (p < 0.05). In measurements conducted at residential properties prior to decontamination and demolition, the median ambient dose equivalent rate measured at house entrances was 0.96 µSv/h. The results of this study demonstrated that the ambient dose equivalent rates on roads measured by car-borne surveys across all Specified Living Areas for Returnees of Okuma Town decreased over the study period. In contrast, the estimated annual external dose at residential properties satisfied the reference level of 20 mSv set by the Ministry of the Environment as a guideline for lifting evacuation orders in most areas, but did not reach the long-term post-accident target level of 1 mSv. These findings provide evidence to inform ongoing discussions on evacuation order policies and long-term radiation management strategies.

## Introduction

Following the 2011 accident at the Tokyo Electric Power Company Fukushima Daiichi Nuclear Power Plant (FDNPP), residents of surrounding municipalities were forced to evacuate for prolonged periods [1]. Even nearly 15 years after the accident, many residents of Okuma Town, Fukushima Prefecture, continue to live outside the town. Okuma Town is the host municipality of the FDNPP, and a full evacuation order was issued immediately after the accident [1].

Since then, evacuation orders have been lifted in a stepwise manner. In 2019, evacuation orders were lifted in parts of the mountainous areas of the town (Ogawara district and Chuyashiki district), and in 2022, evacuation orders were also lifted in the central area of Okuma Town (the Specified Reconstruction and Revitalization Base Areas) [2]. Nevertheless, approximately 50.9% of the town area (4,004 ha) remains designated as difficult-to-return zones (DRZs), and many residents remain evacuated [2]. At present, approximately 1,300 residents live in Okuma Town, corresponding to only about 10% of the pre-accident population [2].

For the long-term recovery of the town, it is important to create conditions under which former residents are able to return if they wish to do so. Achieving this requires not only the restoration of living environments, but also the provision of objective, easily understandable information regarding the radiation environment. Against this background, the Japanese government has established Specified Living Areas for Returnees (SLARs) within DRZs and has progressively implemented decontamination activities and infrastructure development to enable residents with an intention to return to resume their lives [3]. Compared with other DRZs that include extensive forested areas, the SLARs are areas with a higher likelihood of future habitation and therefore occupy an important position from the perspectives of radiation protection and recovery policy [4].

Previous studies have extensively investigated the radiation environment in Fukushima Prefecture following the nuclear accident, including large-scale monitoring of ambient dose equivalent rates and evaluations conducted after the lifting of evacuation orders [1,5,6]. These studies have contributed substantially to understanding regional trends in radiation exposure and the effectiveness of remediation measures. However, few studies have reported evaluations of environmental radioactivity based on field measurements conducted in SLARs located within DRZs prior to the full-scale implementation of decontamination and demolition activities. Moreover, data focusing on prospective living environments, such as public roads and individual residential properties in which residents are expected to spend substantial time after returning, remain scarce.

Against this background, the present study aimed to evaluate environmental radioactivity in the SLARs of Okuma Town based on field measurements conducted before the progression of decontamination and demolition activities. Specifically, we measured ambient dose equivalent rates on public roads using a car-borne survey and within individual residential properties to characterize the spatial distribution of radiation levels and to estimate potential radiation exposures under scenarios of resident returns. By providing baseline data prior to remediation, this study seeks to contribute to the assessment of future dose reduction effects and to support ongoing discussions of radiation protection and recovery planning in post-accident settings.

## Materials and methods

### Survey location

The FDNPP (37° 25’ N, 141° 02’ E) is located across Okuma Town and Futaba Town in Fukushima Prefecture, Japan. Although evacuation orders for Okuma Town were partially lifted in 2019, approximately 50.9% of the town remains designated as DRZs. Within these zones, approximately 440 ha have been designated as SLARs [4].

**Fig 1:**
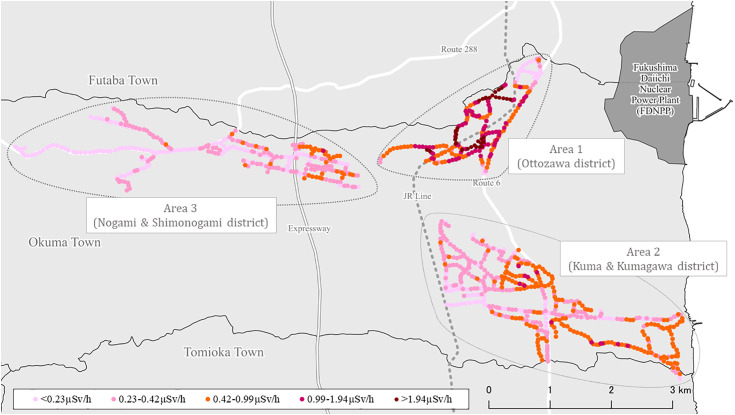
Mapped ambient dose equivalent rate distribution (September 2025). Ambient dose equivalent rate and geographic location data were obtained from the RadiProbe software and replotted by the authors to create this replacement figure on a public-domain base map from the Natural Earth (https://www.naturalearthdata.com/). This figure is similar to, but not identical to, the original RadiProbe software image and is provided for illustrative purposes only. In addition, area lines were added by the authors to indicate the three study areas: Area 1, the Ottozawa district, where little or no decontamination had been conducted; Area 2, the Kuma and Kumagawa districts, where decontamination was ongoing; and Area 3, the Nogami and Shimonogami districts. This figure was created by the authors and is published under the CC BY 4.0 license.

In this study, SLARs adjacent to the Specified Reconstruction and Revitalization Base Areas were divided into three areas according to differences in the status of decontamination (Fig 1): Area 1 (Ottozawa district), in which little to no decontamination had been conducted; Area 2 (Kuma and Kumagawa districts), in which decontamination was ongoing; and Area 3 (Nogami and Shimonogami districts), in which major roads had already been decontaminated following the lifting of the evacuation order.

### Car-borne survey

From November 2024 to September 2025, monthly car-borne surveys were conducted within the SLAR of Okuma Town using the Radi-probe system (Chiyoda Technol Corp., Tokyo, Japan). Field surveys in this study were conducted with permission from the Okuma Town office. A standard passenger vehicle was used, and the survey meter was installed on the front passenger seat at a height of approximately 1 m above ground level.

The system consisted of a CsI spectral survey meter (HDS-101GN; Mirion Technologies, Atlanta, GA, USA), a GPS receiver, a compact camera, and a control PC, enabling real-time acquisition of ambient dose equivalent rates and gamma-ray energy spectra [7].

All measured data were stored together with synchronized GPS information, enabling visualization of spatial dose distributions (Fig 1). To correct attenuation effects caused by the vehicle body, shielding coefficients were applied to the measured ambient dose equivalent rates. The coefficients ranged from 1.20 to 1.68, reflecting variations in measurement conditions such as vehicle structure and detector positioning. During the surveys, the vehicle was driven while attempting to maintain a relatively constant speed. However, variations in speed were unavoidable due to traffic conditions and road environments, which may have introduced minor variability in the measurements. The proportions of dose rate categories were calculated based on the number of measurement data points recorded at fixed time intervals (5 s) during the car-borne surveys. All surveys were conducted along the same routes as far as practical to allow comparison over time. However, measurements were not always obtained at exactly the same locations due to practical constraints during field surveys.

### Residential site measurements

Ambient dose equivalent rate measurements at residential properties were conducted from November 2024 to February 2025. A total of 107 households that had applied for decontamination or demolition in fiscal year 2024 and provided informed consent were included in the study.

A NaI scintillation survey meter was used, and measurements were conducted outdoors at a height of 1 m above ground level at five locations within each property: the front entrance, behind the house, the right side of the house, the left side of the house, and the front yard. The front entrance was selected as a location frequently used by residents, while the other measurement points were placed at the front, back, left, and right sides of each property to assess the radiation environment across the entire premises. For each residential property, summary dose estimates were calculated as the median (minimum–maximum) values across all measurement locations.

### Effective dose calculation

In this study, environmental radiation was evaluated in accordance with the indicators for assessing additional radiation doses provided by the Ministry of the Environment. Accordingly, the annual external effective dose was calculated using the method presented by the Ministry [8].

The annual external effective dose was calculated using the following equations [8]:


$$\begin{array}{c} E=\sum_{i=\text{1}}^{\text{3}\text{6}\text{5}}E_{i} \end{array}$$

E _i_ ＝ E _out_ ＋E _in_

E _out_＝ D ·T_out_

E _in_= D· T _in_· r

where E is the annual external effective dose (mSv); E _i_ is the estimated daily external effective dose (mSv); E _out_ and E _in_ are the outdoor and indoor effective doses; D are ambient dose rates measured at 1 m above ground outdoors (µSv/h); T represents occupancy time (16 h indoors, 8 h outdoors per day), based on the guidelines of the Ministry of the Environment; and r = 0.4 is the gamma-ray attenuation factor for wooden houses [9].

### Data analysis

All car-borne survey data showed non-normal distributions. Differences among measurement areas during the same period and temporal variations within each area were analyzed using the Mann–Whitney U test. Differences among the three areas in residential measurements were analyzed using the Kruskal–Wallis test followed by Dunn’s test for multiple comparisons, since the data exhibited a non-normal distribution. IBM SPSS (version 28; Armonk, NY, USA) was used for all data analyses. Values of p < .05 were considered indicative of statistical significance.

## Results

**Fig 2:**
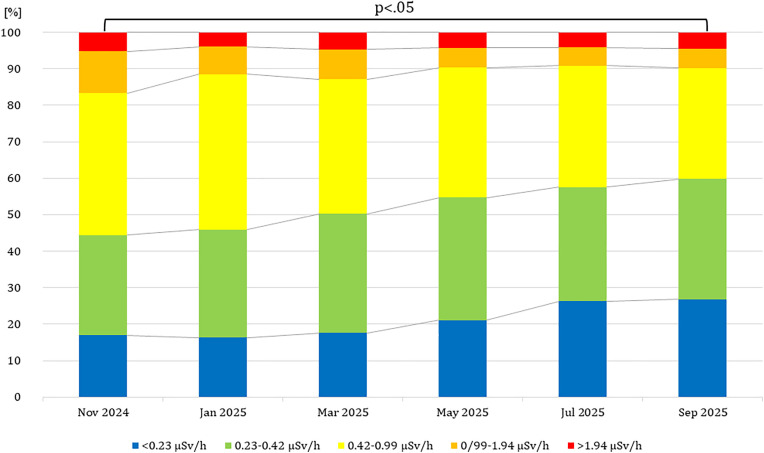
Temporal variation in the ambient dose equivalent rate on public roads in the SLAR.

**Fig 3:**
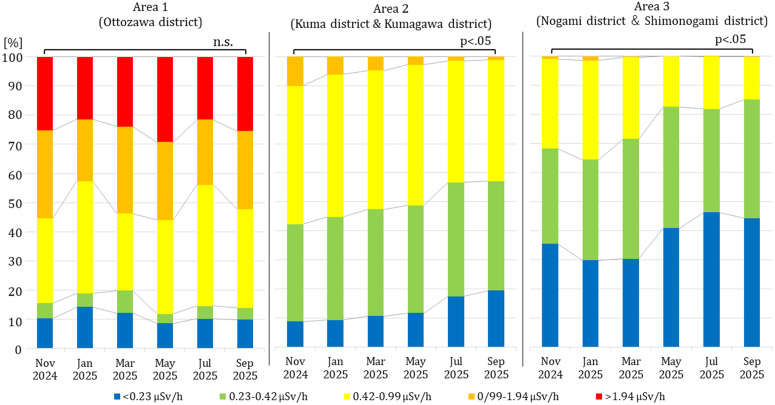
Temporal variation in ambient dose equivalent rate on public roads by area in the SLAR.

The car-borne survey showed that the proportion of locations with an ambient dose rate of 0.42 µSv/h or higher decreased from 55.6% in November 2024 to 40.2% in September 2025 (Fig 2). When analyzed by area, the proportion of locations with an ambient dose rate of 0.42 µSv/h or higher showed a significant decrease over time in Areas 2 and 3. In contrast, no significant temporal change in dose levels was observed in Area 1 (Fig 3).

In September 2025, the median ambient dose equivalent rate on public roads was 0.34 µSv/h. The median ambient dose equivalent rates on public roads were 1.05 µSv/h in Area 1, 0.37 µSv/h in Area 2, and 0.24 µSv/h in Area 3 (Table 1).

**Table 1:** Ambient dose equivalent rates on public roads by area in the SLAR.

	Ambient dose equivalent rate[μSv/h]			
	Median (min-Max)			
Surveydate	All Area	Area1Ottozawa district	Area2Kuma district& Kumagawa district	Area3Nogami district & Shimonogami district
Nov-2024	0.49(0.06-4.90)	1.09(0.08-4.90)	0.49(0.13-1.74)	0.29(0.06-1.35)
Dec-2024	0.45(0.05-4.57)	0.95(0.05-4.48)	0.42(0.13-1.46)	0.34(0.06-1.28)
Jan-2025	0.45(0.06-4.57)	0.85(0.07-4.57)	0.45(0.12-1.92)	0.33(0.06-1.66)
Feb-2025	0.46(0.07-7.02)	1.17(0.08-7.02)	0.47(0.11-2.36)	0.33(0.07-2.34)
Mar-2025	0.42(0.06-4.89)	1.09(0.07-4.89)	0.44(0.12-1.49)	0.31(0.06-1.18)
Apr-2025	0.41(0.06-4.45)	0.96(0.06-4.45)	0.44(0.12-1.38)	0.28(0.07-1.52)
May-2025	0.38(0.06-3.83)	1.13(0.08-3.83)	0.43(0.11-1.31)	0.26(0.06-0.90)
Jun-2025	0.35(0.05-4.23)	0.91(0.06-4.23)	0.38(0.11-1.17)	0.23(0.05-0.96)
Jul-2025	0.36(0.06-4.10)	0.90(0.07-4.10)	0.38(0.11-1.37)	0.24(0.06-1.01)
Aug-2025	0.36(0.05-5.29)	1.00(0.05-5.39)	0.37(0.13-1.09)	0.29(0.06-1.21)
Sep-2025	0.34(0.05-4.68)	1.05(0.07-4.68)	0.37(0.10-1.29)	0.24(0.05-1.05)

Note: Area 1 = Ottozawa district; Area 2 = Kuma & Kumagawa district; Area 3 = Nogami & Shimonogami district

**Fig 4:**
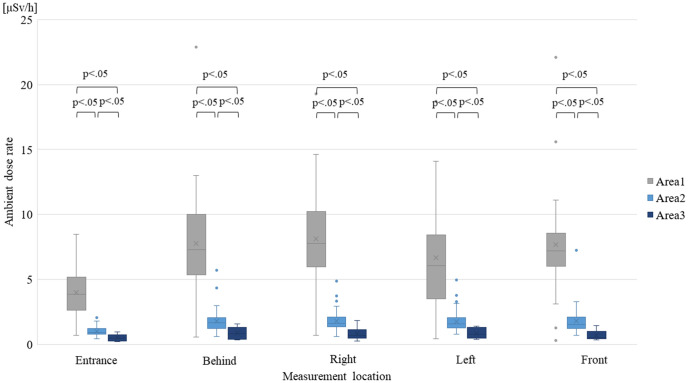
Ambient dose equivalent rates at measurement points in residential properties.

Ambient dose equivalent rates were also measured within the premises of 107 residential properties. The median ambient dose equivalent rate in front of house entrances was 0.96 µSv/h. By area, median ambient dose equivalent rates in front of house entrances were 3.86 µSv/h in Area 1, 0.90 µSv/h in Area 2, and 0.45 µSv/h in Area 3 (Table 2). The median ambient dose equivalent rate of all measurement locations was 1.61 µSv/h, with values of 6.54 µSv/h in Area 1, 1.46 µSv/h in Area 2, and 0.65 µSv/h in Area 3. At all measurement points, including the house entrance, rear side of the house, right side, and left side of the house, ambient dose equivalent rates were highest in Area 1, whereas significantly lower values were observed in Areas 2 and 3 (Fig 4).

**Table 2:** Ambient dose equivalent rates and effective doses at residential properties from November 2024 to February 2025.

	All areas	Area 1	Area 2	Area 3
	n = 107	n = 25	n = 73	n = 9
Ambient dose rate[μSv/h]	Median(min-Max)	Median(min-Max)	Median(min-Max)	Median(min-Max)
All measurement locations	1.61	6.54	1.46	0.65
	(0.22-22.9)	(0.30-22.9)	(0.42-7.24)	(0.22-1.84)
Entrance	0.96	3.86	0.9	0.45
	(0.22-8.48)	(0.67-8.48)	(0.42-2.47)	(0.22-0.99)
Behind	1.79	7.35	1.63	0.96
	(0.34-22.9)	(0.54-22.9)	(0.59-5.68)	(0.34-1.54)
Right	1.8	7.8	1.61	0.68
	(0.23-19.3)	(0.70-19.3)	(0.58-4.84)	(0.23-1.84)
Left	1.7	6.28	1.56	0.9
	(0.39-18.7)	(0.40-18.7)	(0.75-4.84)	(0.39-1.39)
Front yard	1.78	7.36	1.51	0.55
	(0.30-22.1)	(0.30-22.1)	(0.67-7.24)	(0.32-1.44)
Effective dose	8.46	34.37	7.67	3.42
[mSv] ^*1^				

Note: Area 1 = Ottozawa district; Area 2 = Kuma & Kumagawa district; Area 3 = Nogami & Shimonogami district. ^*1^: Effective doses were calculated using the equations in reference [8], based on the median ambient dose equivalent rates from all measurement locations.

## Discussion

In this study, we evaluated ambient dose equivalent rates on public roads using a car-borne survey, as well as ambient dose equivalent rates within residential properties, in the SLAR in Okuma Town. The results demonstrated that the overall proportion of relatively high-dose locations on public roads decreased over the study period (Table 1, Figs 2 and 3). In particular, significant temporal decreases were observed in Areas 2 and 3, whereas no significant change was detected in Area 1 (Table 2, Fig 4). A similar pattern was observed in residential properties, whereambient dose equivalent rates were significantly lower in Areas 2 and 3 compared with Area 1. A comparison between measurements obtained on public roads and those within residential properties enables a more realistic understanding of the radiation environment relevant to returning residents. In this study, ambient dose equivalent rates measured within residential properties tended to be higher than those measured on public roads. This finding may be explained by the fact that radiocesium is more readily removed from paved surfaces through weathering processes such as rainfall and surface runoff, whereas higher dose rates tend to persist in residential and soil-covered areas.

Furthermore, a consistent spatial pattern was observed in both road-based and residential measurements, with higher dose levels in Area 1 and lower levels in Areas 2 and 3. This consistency suggests that relative differences in radiation levels among administrative areas are maintained even across different measurement environments. These findings support existing knowledge regarding spatial heterogeneity in the post-accident radiation environment and provide important field-based data for assessing radiation exposure in areas in which the progress of decontamination varies. These findings indicate that, even within the SLARs, the radiation environment is not homogeneous and that substantial differences exist among administrative areas in terms of dose distribution and the progress of dose reduction [1,5]. In Areas 2 and 3, decontamination activities and building demolition have already been initiated, and the dose reduction effects of these measures may have influenced the observed dose levels [1,6]. This spatial heterogeneity has important implications for radiation protection and reconstruction efforts. Rather than applying uniform countermeasures across the entire designated area, considering area-specific approaches based on the local radiation environment may be reasonable [10,11]. In areas where dose levels have relatively decreased, it may be possible to advance measures aimed at supporting the return of residents and the restoration of daily life, such as the relaxation of access restrictions, whereas areas with persistently higher dose levels may require continued environmental management and monitoring [12,13].

SLARs were established based on the intentions of residents to return, with priority given to those administrative areas in which a higher proportion of residents expressed a desire to resume living. Compared with other DRZs that include extensive forested land, these designated areas are therefore more likely to be inhabited in the future. The present results, demonstrating reductions in ambient dose equivalent rates in parts of the designated area, provide scientific information that may help inform ongoing assessments and discussions on appropriate radiation protection and recovery planning in post-accident settings [10].

At the same time, previous studies have suggested that reductions in radiation dose alone do not necessarily lead directly to residents’ decisions to return. Even when levels decrease, decisions regarding return are influenced by multiple factors, including the restoration of infrastructure, access to medical and welfare services, educational environments, and employment opportunities [14,15]. Accordingly, radiation dose management should be implemented as part of comprehensive support packages that integrate infrastructure development and social support measures [16,17].

In this study, ambient dose equivalent rates at front entrances tended to be lower than those at other measurement points within residential properties. This is likely because front entrances are often covered with concrete or other impermeable surfaces. Previous studies have shown that radioactive cesium deposited on impermeable surfaces such as roads and roofs is efficiently removed by rainfall and surface runoff, leading to faster decreases in ambient dose rates compared with soil-covered areas [13]. In contrast, most other measurement points were located on soil-covered ground, where radioactive cesium tends to be retained for longer periods, resulting in relatively higher ambient dose equivalent rates.

Notably, the present study also identified locations in Area 1 where ambient dose equivalent rates remained relatively high, even approximately 15 years after the accident. This finding suggests that time-dependent decay and general remediation measures alone may be insufficient to achieve dose reduction in certain locations [1,18]. Continued monitoring focused on such localized higher-dose areas, together with targeted environmental management, remains important for long-term radiation protection and recovery [11,12].

This study has several limitations. First, ambient dose equivalent rates within residential properties were measured at a limited number of points within each property, and these measurements thus do not necessarily adequately represent the status of entire premises or future residential environments. In particular, when residents return in the future, existing houses are expected to be demolished, followed by decontamination of the land. The rates measured in this study thus do not directly reflect radiation exposure levels during future residential use. Nevertheless, the measurements taken in the course of this study provide valuable empirical data on the radiation environment prior to demolition and decontamination. These pre-remediation measurements provide an important baseline for evaluating changes in ambient dose equivalent rates before and after remediation activities. Moreover, site-specific measurements based on actual field surveys contribute to the characterization of localized dose distributions that may not be fully captured by model-based assessments alone [18]. In addition, annual external effective doses in this study were evaluated using the calculation method adopted by the Ministry of the Environment, Japan for regulatory and administrative purposes. This method treats ambient dose rates as a proxy for external effective dose and does not apply an effective dose conversion coefficient. Although calculations based on ICRP Publication 144 were not performed in the present study, such approaches may be useful for more realistic effective dose assessments [19].. Further, the measurement period of this study was relatively short and did not account for seasonal or long-term temporal variations in ambient dose equivalent rates. Previous studies have shown that ambient dose rates can change over time due to factors such as weather conditions, radioactive decay, and environmental processes [20]. As a result, continuous or long-term monitoring would be necessary to better understand temporal trends and improve long-term dose assessments.

## Conclusions

This study evaluated ambient dose equivalent rates on public roads and within residential properties in SLAR in Okuma Town, Fukushima Prefecture, to assess potential radiation exposure under scenarios of resident return. The results showed an overall decreasing trend in ambient dose equivalent rates on public roads during the study period.

In contrast, estimated external dose within residential properties prior to remediation and demolition remained below the reference level of 20 mSv for post-accident exposure situations, while indicating the continued importance of remediation and other dose reduction measures. As demolition and decontamination activities are currently ongoing in the study area, continued dose assessments will be essential to support the optimization of radiation protection and to provide a scientific basis for future recovery and reconstruction efforts.

## Supporting information

S1 File The car borne survey. (XLSX)

S2 File Residential properties. (XLSX)

## References

[1] United Nations Scientific Committee on the Effects of Atomic Radiation UNSCEAR. Sources, effects and risks of ionizing radiation. New York: United Nations; 2014.

[2] Reconstruction Agency. Current status and initiatives of Okuma Town. Accessed 2025 December 22. https://www.reconstruction.go.jp/files/user/topics/main-cat7/sub-cat7-2/20240913_03.pdf

[3] Reconstruction Agency. Current status of reconstruction and future efforts. https://www.reconstruction.go.jp/files/user/english/topics/Progress_to_date/English_August_2024_genjoutorikumi-E.pdf

[4] About the specified reconstruction and revitalization base areas and the specified living areas for returnees. Accessed 2025 December 22. https://www.pref.fukushima.lg.jp/site/portal-english/en-1-3-3.html

[5] Kobayashi S, Shinomiya T, Kitamura H, Ishikawa T, Imaseki H, Oikawa M, et al. Radioactive contamination mapping of northeastern and eastern Japan by a car-borne survey system, Radi-Probe. J Environ Radioact. 2015;139:281–93. doi: 10.1016/j.jenvrad.2014.07.026 25189817

[6] Yasutaka T, Naito W. Assessing cost and effectiveness of radiation decontamination in Fukushima Prefecture, Japan. J Environ Radioact. 2016;15:512–20. doi: 10.1016/j.jenvrad.2015.05.012 26051754

[7] Mirion T. Handheld search and isotope identification device. Accessed 2025 December 22. https://mirionprodstorage.blob.core.windows.net/prod-20220822/cms4_mirion/files/pdf/spec-sheets/spc-174-en-a_hds101g-gn.pdf?1645216852

[8] Ministry of the Environment of Japan. Additional exposure doses after an accident (example of calculation). Accessed 2025 December 22. https://www.env.go.jp/en/chemi/rhm/basic-info/1st/02-04-11.html

[9] Ministry of the Environment of Japan. Shielding and reduction coefficient. Accessed 2025 December. https://www.env.go.jp/en/chemi/rhm/basic-info/1st/02-04-10.html

[10] Lochard J, Bogdevitch I, Gallego E, Hedemann-Jensen P, McEwan A, Nisbet A, et al. ICRP Publication 111 - Application of the Commission’s recommendations to the protection of people living in long-term contaminated areas after a nuclear accident or a radiation emergency. Ann ICRP. 2009;39(3):1–4, 7–62. doi: 10.1016/j.icrp.2009.09.008 20472181

[11] Oughton D. Societal and ethical aspects of the Fukushima accident. Integr Environ Assess Manag. 2016;12(4):651–3. doi: 10.1002/ieam.1831 27640410

[12] The 2007 Recommendations of the International Commission on Radiological Protection. ICRP publication 103. Ann ICRP. 2007;37(2–4):1–332. doi: 10.1016/j.icrp.2007.10.003 18082557

[13] Yoshimura K. Air dose rates and cesium-137 in urban areas — deposition, migration, and time dependencies after nuclear power plant accidents. J Nucl Sci Technol. 2022;59(1):25–33.

[14] Lochard J, Schneider T, Ando R, Niwa O, Clement C, Lecomte JF. An overview of the Fukushima dialogue initiative process. Radioprotection. 2019;54(2):87–101.

[15] Tsubokura M, Kato S, Nihei M, Sakuma Y, Furutani T, Uehara K, et al. Limited internal radiation exposure associated with resettlements to a radiation-contaminated homeland after the Fukushima Daiichi nuclear disaster. PLoS One. 2013;8(12):e81909. doi: 10.1371/journal.pone.0081909 24312602 PMC3846705

[16] Bromet EJ. Emotional consequences of nuclear power plant disasters. Health Phys. 2014;106(2):206–10. doi: 10.1097/HP.0000000000000012 24378494 PMC3898664

[17] Lochard J, Wynne B. Radiological protection at the service of society. Annals of the ICRP. 2012;41(3–4):84–90.

[18] Hashimoto S, Ugawa S, Nanko K, Shichi K. The total amounts of radioactively contaminated materials in forests in Fukushima, Japan. Sci Rep. 2012;2:416. doi: 10.1038/srep00416 22639724 PMC3360326

[19] Petoussi-Henss N, Satoh D, Endo A, Eckerman KF, Bolch WE, Hunt J, et al. ICRP Publication 144: dose coefficients for external exposures to environmental sources. Ann ICRP. 2020;49(2):11–145. doi: 10.1177/0146645320906277 33115250

[20] Taira Y, Matsuo M, Orita M, Matsunaga H, Kashiwazaki Y, Xiao X. Regional case studies: Environmental radioactivity levels and estimated radiation exposure doses of residents and workers in areas affected by the Fukushima Daiichi Nuclear Power Plant accident. Radiat Environ Med. 2023;12(1):37–52.

